# Cerebral Small Vessel Disease in Elderly Patients with Sudden Sensorineural Hearing Loss

**DOI:** 10.1097/MAO.0000000000003813

**Published:** 2023-01-21

**Authors:** Fieke K. Oussoren, Roeland B. van Leeuwen, Tjard R. Schermer, Louise N. F. Poulsen, Joost J. Kardux, Tjasse D. Bruintjes

**Affiliations:** ∗ Apeldoorn Dizziness Centre, Gelre Hospitals, Apeldoorn, The Netherlands; † Department of Otorhinolaryngology, Leiden University Medical Center, Leiden, The Netherlands; ‡ Department of Primary and Community Care, Radboud Institute for Health Sciences, Radboud University Medical Center, Nijmegen, The Netherlands; § Department of Radiology, Gelre Hospitals, Apeldoorn, The Netherlands

**Keywords:** MRI, Neurotology, SSNHL, Stroke

## Abstract

The cause of sudden sensorineural hearing loss (SSNHL) is uncertain in a significant number of patients. A vascular etiology has been proposed because SSNHL increases the risk of developing a stroke. Cardiovascular risk factors can cause cerebral small vessel disease (CSVD). The presence of CSVD in turn raises the risk of stroke.

**Aim:**

The aim of this study was to compare the presence of CSVD and cardiovascular risk factors in elderly patients with idiopathic SSNHL (iSSNHL) to a control cohort.

**Method:**

Patients with iSSNHL of 50 years and older were compared with a control cohort with patients suspected of trigeminal neuralgia or vestibular paroxysmia. The primary outcome was the difference in the number of white matter hyperintensities using the ordinal Fazekas scale. Secondary outcomes were the presence of brain infarctions on MRI and the difference in cardiovascular risk factors.

**Results:**

In the SSNHL cohort, Fazekas score 2 was most frequently seen compared with Fazekas 1 in the control cohort. The distribution of Fazekas scores did not differ significantly. The sum of the Fazekas scores were 13,925 and 14,042 for iSSNHL and controls, respectively (*p* = 0.908). Brain infarctions were seen in 8 patients with iSSNHL (n = 118) and in 13 patients in the control cohort (n = 118) (*p* = 0.361). None of the cardiovascular risk factors were more frequently seen in the iSSNHL cohort.

**Conclusion:**

Patients with iSSNHL did not exhibit more CSVD on MRI than controls. This result is in contrast with previous literature demonstrating a higher risk of stroke in patients with iSSNHL than in controls. A prospective analysis with a larger study population is therefore warranted.

## INTRODUCTION

Sudden sensorineural hearing loss (SSNHL) is an otological emergency in which an evident cause can be identified in 7% to 47% of the cases (1–3). Reported incidence rates range from 11 to 77 per 100,000 people each year (4). The current treatment, which is mainly based on a viral genesis with high-dose corticosteroid therapy, seems to be insufficient in 39% to 75% of patients (5). For these two reasons, a different etiology and subsequent treatment of SSNHL should be considered in several patients. Besides viral cochleitis, the following entities have been proposed to cause SSNHL: ototoxic drugs, neoplasms, autoimmune disorders, and microvascular disease (2).

Especially in the last two decades, research has focused on the hypothesis of a vascular origin (6–8). Hearing loss or vestibular loss is the sole manifestation in 0.6% to 3% of patients with infarction of the posterior cerebral circulation (9), whereas hearing or vestibular loss was succeeded by neurological symptoms in 8% to 31% (9). A recent meta-analysis demonstrated a 1.46 higher risk of stroke in patients with idiopathic SSNHL (iSSNHL) (10). Sudden deafness might therefore be an indicator of cerebrovascular disease and not merely a local condition.

Cerebral small vessel disease (CSVD) is a chronic progressive disorder of the arterioles, capillaries, and small veins supplying the cerebral white matter. Some uncommon genetic or infectious forms aside, CSVD is mainly associated with cardiovascular risk factors, in particular increasing age and hypertension and, therefore, a major cause of stroke and vascular dementia (11–14). Characteristics of CSVD are white matter hyperintensities (WMHs), lacunes of presumed vascular origin, cerebral microbleeds, and brain infarctions (15–18). The presence of WMH alone results in a threefold risk of stroke (19). Fusconi et al. investigated the presence of WMH on MRI imaging in patients with iSSNHL. Overall, the difference in WMH was not significant compared with the general population (20). However, individuals with iSSNHL between 48 and 60 years old did have a 26% higher probability of WMH on MRI (20).

Research on the vascular involvement of iSSNHL in elderly patients is limited. This retrospective case-controlled study aimed at ascertaining whether, compared with a control cohort of patients without hearing loss, more WMHs and infarctions are seen on MRI imaging in elderly patients with iSSNHL, 50 years or older.

## METHODS

### Setting

This retrospective case-controlled study was based on hospital records from patients either visiting the outpatient otorhinolaryngology or neurology departments of Gelre Hospitals Apeldoorn and Zutphen or the Apeldoorn Dizziness Centre (ADC), located in Gelre Hospitals. The ADC serves as a tertiary referral center that specializes in the diagnostic and therapeutic workup of dizziness. It is a multidisciplinary center involving the Neurology, Clinical Neurophysiology, and Otorhinolaryngology departments of the Gelre Hospitals Apeldoorn. This retrospective cohort study was approved by the institutional review board at Gelre Hospitals Apeldoorn.

### Inclusion

All records of patients seen by an otolaryngologist with acute hearing loss from January 2010 until May 2022 were retrieved. The definition of iSSNHL used in this study is a rapidly developing sensorineural hearing loss with a minimum of 30 dB over at least three contiguous frequencies on tone audiometry that occurs within a period of 72 hours (5,21–23), in the absence of an identifiable cause for the hearing loss, like Menière’s disease, ototoxic drugs, etc. Patients with iSSNHL were included in the study cohort if they had received an MRI to exclude a cerebellopontine neoplasm.

A control cohort was compiled of patients who either visited the outpatient neurological department with facial pain, suspected for trigeminal neuralgia, or visited the ADC with recurrent episodes of spontaneous vertigo lasting several seconds, suggestive of vestibular paroxysmia. All patients received an MRI cerebrum to rule out the presence of an intracranial neoplasm or to detect a neurovascular conflict. No association between these diseases and CSVD has been described. Subjects in the study and control cohorts were matched for age and gender, with a maximum age difference of 1 year.

Exclusion criteria were 49 years or younger and a history of cerebrovascular accident or transient ischemic attack.

### MRI Protocol

An MRI was suitable for the radiological assessment of WMHs and brain infarctions if at least one sequence of the entire brain, either FLAIR or T2, was available. Data on the MRI sequences used are displayed in Table 1. All imaging was performed using a 1.5-T MRI scanner. The cerebral sequence was depicted with a slice thickness of 5 mm in the axial plane, with the exception of one case witch a slice thickness of 4 mm.

**TABLE 1:** The mode (min and max repetition time and echo time) per scanning protocol characteristic of 88 FLAIR MRI scans and 148 T2 scans

Sequence	TR (ms)	TE (ms)	Slice Thickness (mm)
FLAIR	8,000 (6,000, 8,000)	86 (86,159)	5
T2	4,620 (2,073, 5,880)	89 (82,120)	5

TE, echo time; TR, repetition time.

### Outcomes

**FIG. 1:**
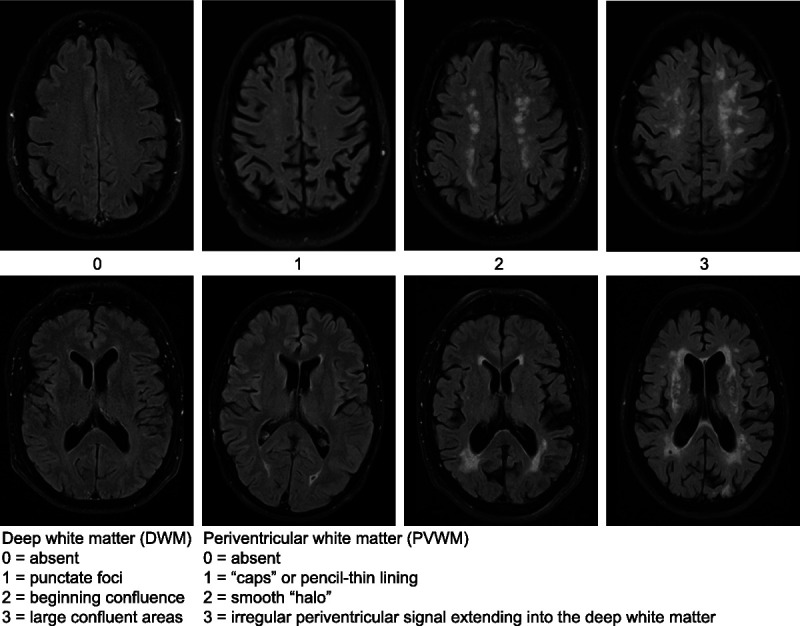
Fazekas scale for MRI imaging. The figure displays hyperintensities in the deep white matter (*upper row*) and periventricular white matter (*lower row*) (24).

The primary outcome was the degree of cerebrovascular damage assessed on MRI imaging by measuring the Fazekas score. The Fazekas score is a validated diagnostic tool for assessing the severity of white matter hyperintensities in both the periventricular and the deep white matter with a possible score from 0 to 6, where 0 means no hyperintensities present (see Figure 1).

The secondary outcome was the presence of brain infarctions on MRI imaging. Brain infarctions were defined by lesions of the brain of at least 3 mm with a cerebrospinal fluid appearance in gray intensity on both a FLAIR and a T2 MRI sequence and differentiable from leukoaraiosis and dilated Virchow–Robinson spaces (25).

The following cardiovascular risk factors were identified: smoking, hypertension, hyperlipidemia, diabetes, myocardial infarction history, and atrial fibrillation. Hypertension and diabetes were defined as being present either by having a positive medical history or if medications for these conditions were used. In case no complete medical history had been obtained, the variable was defined as missing. Hyperlipidemia was defined as being present when the patient had a positive medical history of dyslipidemia, used statins, or had an elevated total cholesterol level of >4.9 mmol/L within a month before or after presentation at the outpatient dizziness center, neurology department, or otorhinolaryngology department.

#### Assessment

MRI imaging was assessed by two neuroradiologists separately, LP and JK. The two radiologists involved in this study have multiple years of experience in examining MRI imaging of the head and neck. To limit observer bias, both radiologists were blinded for the patients’ characteristics or study arm.

If there was a difference in Fazekas rating between the two raters, the following rules were applied. If the difference was one rank, the highest rank was then used. If the difference was two ranks or more, the radiologists reviewed the case together until consensus was reached.

Baseline characteristics, data from diagnostic tests, and MRI ratings were gathered and deidentified in a Castor electronic database (CastorECD, Amsterdam, The Netherlands).

#### Rater Reliability Testing

Inter- and intrarater reliability for the two radiologists was assessed for the Fazekas rating scale. All retrieved MRI scans were rated by both raters independently. Sample size calculation showed that 30 patients had to be rated twice by both raters to evaluate the intrarater reliability. A weighted Cohens’ *κ* coefficient was calculated using linear weighting, where the difference between low and high ratings is of equal importance.

#### Statistical Analysis

Continuous variables were described using the following summary descriptive statistics: number of nonmissing values, mean and standard deviation in case of normally distributed data, or median and interquartile ranges in case of nonnormally distributed data.

Categorical variables were described using frequencies and percentages. Percentages were calculated on the number of nonmissing observations.

The 95% confidence intervals were calculated when applicable. Statistical testing was performed two-sided at a 0.05 significance level.

Differences in the ordinal ranking of the Fazekas scale between the two cohorts were calculated using the Mann–Whitney *U* test for ordinal nonpaired data. The presence of brain infarctions was compared between the cohorts using the χ^2^ test.

A nonparametric independent ordered samples analysis, the Jonckheere–Terpstra test, was performed to compare the mean hearing thresholds at presentation between the different Fazekas scores for each of the following frequencies: 500, 1,000, 2,000, 4,000, and 8,000 Hz.

Ordinal logistic regression analysis was performed to compare the outcomes between the cohorts while adjusting for the potential confounders: study cohort; age; gender; MRI sequence used for assessment; repetition time; echo time; Fletcher index at presentation; average hearing loss in the frequencies 2000, 4,000, and 8,000 Hz; corticosteroid therapy; and hearing improvement.

## RESULTS

A total of 118 patients with iSSNHL were included. The age- and gender-matched control cohort consisted of 79 patients (66.9%) suspected of trigeminal neuralgia and 39 patients (33.1%) suspected of vestibular paroxysmia.

Baseline characteristics of all patients are displayed in Table 2. Both cohorts consisted of more men than women.

**TABLE 2:** Patient characteristics

	SSNHL (N = 118)	Control (N = 118)	Missing	p
Age, mean (SD)	65 (9.3)	65 (9.0)	0	0.491
Gender			0	1.000
Female	52	52		
Male	66	66		
History of myocardial infarction	13 (11.0)	7 (5.9)	0	0.242
Anticoagulant use	21 (17.8)	13 (11.0)	0	0.097
Smoking			34	0.850
Yes	13 (13.7)	12 (11.2)		
Former	13 (13.7)	14 (13.1)		
No	69 (72.6)	81 (75.7)		
Hypertension	43 (38.4)	45 (38.5)	7	1.000
Hyperlipidemia	35 (33.3)	34 (29.1)	14	0.562
Diabetes	9 (7.8)	13 (11.0)	1	0.695
Atrial fibrillation	8 (7.0)	2 (1.7)	3	0.057

Patient characteristics of 118 patients experiencing SSNHL and a control cohort of 118 patients displayed in numbers and percentages. Percentages are calculated based on the number of nonmissing values.

SD, standard deviation; SSNHL, sudden sensorineural hearing loss.

In the iSSNHL cohort, the high fletcher index, the average hearing loss in patients with iSSNHL over the frequencies 1,000, 2,000, and 4,000 Hz was 70 ± 24 dB. One hundred patients (84.7%) received corticosteroid therapy. The other patients either presented at the ENT department outside the therapeutic window for corticosteroid use or declined this therapy of their own accord. Hearing loss improved with at least 20% in 52 patients (44.1%), whereas the hearing threshold did not improve or even worsened over time in 66 patients (55.9%).

### Rater Reliability

All retrieved MRI scans, before age and gender matching, were used for the rater reliability testing. Three hundred and twenty-eight MRI scans were reviewed by both raters. In 201 cases, both radiologists gave the same Fazekas score; in 103 cases, they differed 1 point; and in 24 cases, the difference in score given was 2 points or more. This resulted in a *κ* coefficient of 0.74 for interrater reliability.

Thirty subjects were rated twice by each rater, which resulted in a weighted *κ* coefficient of 0.80 and 0.82 for raters 1 and 2, respectively, suggesting a near-perfect agreement for each rater.

### Fazekas Score

**FIG. 2:**
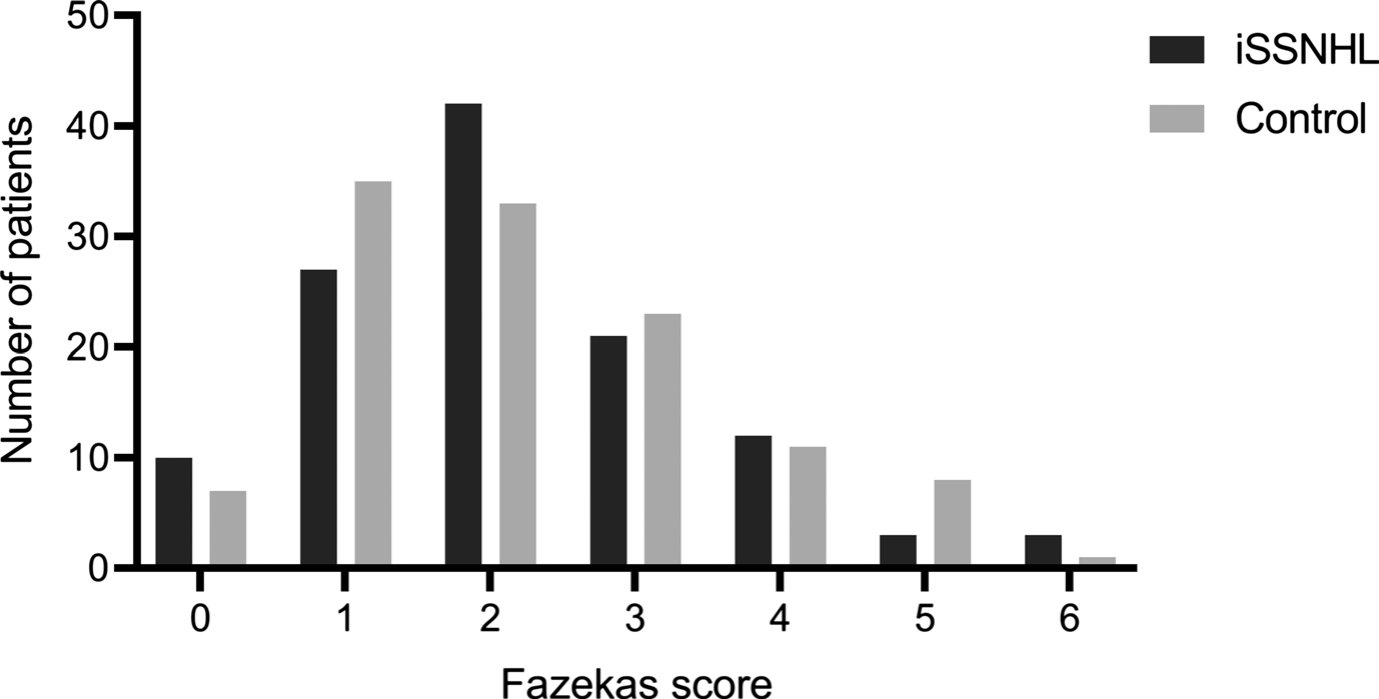
Distribution of Fazekas scores. Fazekas score two was most frequently seen in the iSSNHL cohort compared with Fazekas score 1 in the control cohort. A Mann–Whitney *U* test resulted in a *p* value of 0.908, demonstrating a nonsignificant difference in Fazekas score distribution.

In the SSNHL cohort, Fazekas score 2 was most frequently seen on MRI compared with Fazekas score 1 in the control cohort, see Figure 2. The distribution of the ordinal scale across both cohorts did not differ significantly. The sums of the Fazekas scores were 13,925 and 14,042 for iSSNHL and controls, respectively (*p* = 0.908).

#### Brain Infarctions

Brain infarctions were present in 8 patients (6.8%) with iSSNHL and in 13 patients (11.0%) from the control cohort. The difference between the two cohorts was not statistically significant (*p* = 0.361). In both cohorts, the brain infarctions were most frequently located in the deep white matter.

#### Ordinal Regression Analysis

Table 3 displays the estimates of the odds ratio from the ordinal logistic regression analysis. The variables age, gender, and average hearing loss at presentation significantly increased the risk of receiving a higher Fazekas score. In the multivariate regression analysis, the variables age and female gender increased the risk of CSVD significantly.

**TABLE 3:** Ordinal regression analysis

	Univariate			Multivariate		
	Odds	Sig	95% CI	Odds	Sig	95% CI
SSNHL	1.009	0.908	0.867–1,174			
Age	1.138	0.000	1.105–1.172	1.123	0.000	1.081–1.166
Gender	1.688	0.027	1.063–2.681	1.858	0.040	1.028–3.360
FLAIR sequence	1.221	0.406	0.762–1.956			
Repetition time	1.000	0.776	1.000–1.000			
Echo time	0.996	0.670	0.976–1.016			
Fletcher index	1.017	0.002	1.006–1.028	1.009	0.121	0.998–1.021
Corticosteroid use	1.849	0.184	0.746–4.584			
Hearing improvement	1.227	0.538	0.640–2.355			

Outcomes of the ordinal regression analysis. Displayed are estimated odds, its significance, including lower and upper bound, of receiving a higher Fazekas score when having a 1 year older age, female gender, FLAIR sequence used in MRI assessment, a one point higher repetition time, a one point higher echo time, the high Fletcher index at presentation, receiving corticosteroid use, hearing improvement compared with remaining hearing loss and the study arm.

Sig, significance; FLAIR, fluid attenuated inversion recovery; SSNHL, sudden sensorineural hearing loss.

**FIG. 3:**
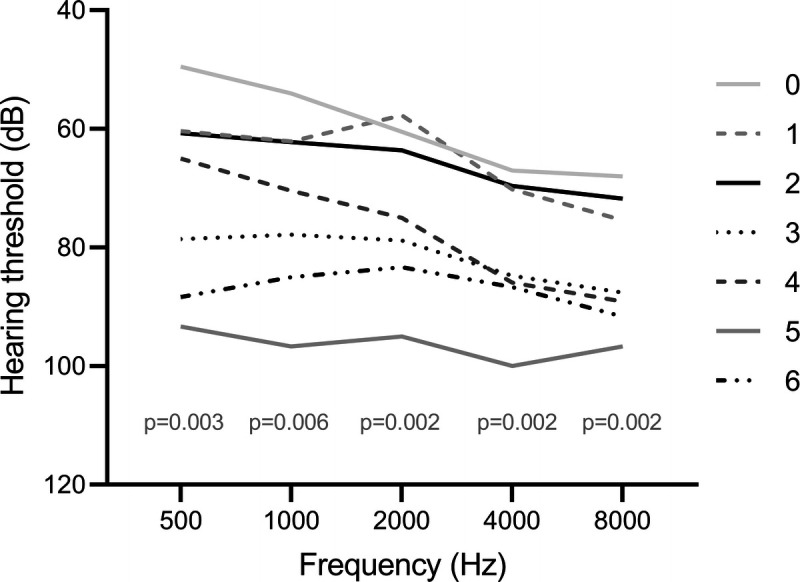
Fazekas scores and mean hearing threshold in dB for each frequency are displayed. Outcomes of the Jonckheere–Terpstra test for each frequency are displayed after *p* in the graft.

When the hearing threshold was analyzed within the iSSNHL cohort, we found a correlation of an increasing Fazekas score and a higher hearing threshold, measured in decibels (see Figure 3). A Jonckheere–Terpstra test estimated this correlation to be statistically significant on all frequencies. This correlation is likely explained by the differences in age because the significant correlation disappeared in the multivariate regression analysis. No correlation was found between the Fazekas score and hearing improvement.

## DISCUSSION

To investigate a possible correlation between CSVD and iSSNHL that could indicate a vascular involvement in the pathogenesis of acute hearing loss in elderly, we compared the degree of WMHs and the presence of brain infarctions between two cohorts. We did not find a significant difference in Fazekas score or presence of brain infarctions in elderly patients with iSSNHL compared with controls.

The outcomes in this study are in contrast with previous literature that demonstrated an increased Fazekas score in elderly patients with iSSNHL (26,27). To date, three studies investigated the association between iSSNHL and CSVD. Ciorba et al. (28) studied the presence of leukoaraiosis in 64 patients with iSSNHL using the Fazekas and Wahlund scale. They did not find a difference in the degree of leukoaraiosis when compared with a control cohort (28). However, patients with a higher Fazekas score did have a lower probability of complete hearing recovery. By contrast, Dicuonzo et al. (27) found significantly more WMHs in both the deep white matter and the periventricular white matter in patients with iSSNHL compared with controls. However, with only 36 subjects in the study sample, a reliable regression analysis could not be performed. Fusconi et al. (25) found a significant correlation between iSSNHL and the Fazekas score among patients 48 to 60 years old; this group only comprised 22% of the study population.

In the elderly population, the prevalence of CSVD ranges from 56% in patients 60 to 70 years old to 74% in 80 years and older (29). This likely explains the relatively high median Fazekas score of 2 in our cohort compared with previous literature. We did not find a difference in distribution of Fazekas scores between both cohorts, nor did we find a correlation between the Fazekas score and the probability of hearing recovery. We did find a correlation between the Fazekas score and the hearing threshold at presentation, as did Ciorba et al., though this difference was caused by increasing age of the subjects (28).

Several authors have investigated the presence of cardiovascular risk factors in patients with iSSNHL. The association between iSSNHL and cardiovascular risk factors, however, remains controversial. In some studies, elevated triglycerides and total cholesterol levels, as well as overweight, diabetes, and a history of cardiovascular events have been associated with an increased prevalence of iSSNHL (30–33), whereas other authors found contradicting results (34). In a recent meta-analysis, Simões et al. (35) summarized that increased total cholesterol levels and hypertriglyceridemia are more frequently present in patients with iSSNHL compared with controls, whereas diabetes and hypertension are not. There was, however, large heterogeneity between published studies, resulting in a small number of studies used in the analysis (35). We found higher prevalences of a medical history with myocardial infarction, anticoagulant use, smoking, and hyperlipidemia in the iSSNHL cohort than in the control cohort, but none of these differences were statistically significant.

Nevertheless, the hypothesis of vascular compromise in the onset of iSSNHL is biologically plausible. Sudden deafness resembles cardiovascular disease like myocardial infarction and stroke in acute and mostly unilateral presentation (1). Also, the cochlea is supplied by a terminal artery without collateral circulation and would therefore be vulnerable to hypoxia (36).

It has been hypothesized that iSSNHL is either due to acute occlusion somewhere along the anterior inferior cerebellar artery and its peripheral branches or caused by blood pressure dysregulation (BPD) (37).

Tange (36) provided a theoretical diagram of the vestibulocochlear blood supply and proposed a classification of four types of vascular inner ear obstruction based on the location of occlusion and its clinical presentation. For example, the partition of the ramus cochlearis could lead to SSNHL in the frequencies 2000 Hz and above without vertigo because this artery is responsible for the supply of the lower part of the cochlea toward the basal turn (36). Unfortunately, in clinical practice, this differentiation in presentation is difficult to establish.

The other hypothesis of vascular involvement in the onset of iSSNHL is BPD. BPD arises when, on the one hand, fibrous hyalinosis causes the tunica adventitia to thicken and lose fibromuscular cells, resulting in a decreased adrenergic regulation, and on the other hand, the lumen of the vessel is decreased because of arteriosclerosis. BPD can thus cause permanent damage to nerve fibers (20).

When these hypotheses prove to be accurate, this could mean that patients with iSSNHL have a vascular compromised brain. This might have serious consequences because CSVD is known to triple the risk of stroke (19,38). Consequently, Lin et al. (39) were the first to report an increased risk of a cerebrovascular accident after experiencing iSSNHL. Several other authors confirmed this increased risk, although all these studies were performed retrospectively (39–42). Further prospective controlled analyses of cardiovascular risk factors, cerebrovascular damage, and risk of stroke in patients with iSSNHL would be appropriate to assess whether our results are accurate and the possible association in elderly is negligible, or if there is indeed an association and if elderly patients with iSSNHL should receive cardiovascular risk management including anticoagulants.

We have to address some limitations of this study. Most importantly, we did not perform a sample size calculation before this study. In the general population, Fazekas distributions are rarely described per age category. And if so, the difference in expected distribution for iSSNHL would remain completely arbitrary. Because of the ordinal character of the variable Fazekas score with 7 possible categories, slight changes in estimated Fazekas distribution could result in very large differences in sample size. Hence, we decided to evaluate the total number of patients we could retrieve from hospital records. A type II error is, therefore, a remote possibility. Consequently, the study is of an exploratory nature. A total population of 118 per study cohort is however in line with other similar studies.

Second, the MRI sequence that was used for assessment was nonuniform. Usually, an MRI cerebrum is performed in patients suspected of trigeminal neuralgia, whereas in the case of iSSNHL and vestibular paroxysmia, an MRI of the cerebellopontine angle is performed with only a single sequence of the entire brain, either T2 or FLAIR. Also, per MRI protocol, the repetition time and the echo time differ. In the regression analysis we demonstrated that the type of MRI sequence, the repetition time, and the echo time did not influence the assigned Fazekas score.

Finally, we did not include patients in whom hearing loss was accompanied by vertigo because in our dizziness center, this combination of symptoms is diagnosed as labyrinthitis. In previous literature, patients experiencing combined hearing loss and vertigo have been included in the iSSNHL group. This is relevant because combined hearing loss and vertigo have an increased risk of stroke compared with ISSNHL or vertigo alone (43).

In conclusion, we did not find an association between SSNHL and CSVD. We did find a higher prevalence of some cardiovascular risk factors in patients with iSSNHL compared with controls, although nonstatistically significant. Further prospective controlled research with larger populations is needed to clarify the vascular involvement in the etiology of iSSNHL.
